# Arrhythmokinesis is evident during unimanual not bimanual finger tapping in Parkinson’s disease

**DOI:** 10.1186/s40734-015-0019-2

**Published:** 2015-04-02

**Authors:** Megan H Trager, Anca Velisar, Mandy Miller Koop, Lauren Shreve, Emma Quinn, Helen Bronte-Stewart

**Affiliations:** Department of Neurology and Neurological Sciences, Stanford University, 300 Pasteur Drive, Stanford, CA 94305 USA; Department of Neurosurgery, Stanford University, Stanford, CA USA

**Keywords:** Parkinson’s disease, Finger tapping, Rhythmicity, Arrhythmokinesis, Quantitative Digitography

## Abstract

**Background:**

Arrhythmokinesis, the variability in repetitive movements, is a fundamental feature of Parkinson’s disease (PD). We hypothesized that unimanual repetitive alternating finger tapping (AFT) would reveal more arrhythmokinesis compared to bimanual single finger alternating hand tapping (SFT), in PD.

**Methods:**

The variability of inter-strike interval (CV_ISI_) and of amplitude (CV_AMP_) during AFT and SFT were measured on an engineered, MRI-compatible keyboard in sixteen PD subjects off medication and in twenty-four age-matched controls.

**Results:**

The CV_ISI_ and CV_AMP_ of the more affected (MA) and less affected (LA) sides in PD subjects were greater during AFT than SFT (P < 0.05). However, there was no difference between AFT and SFT for controls. Both CV_ISI_ and CV_AMP_ were greater in the MA and LA hands of PD subjects versus controls during AFT (P < 0.01). The CV_ISI_ and CV_AMP_ of the MA, but not the LA hand, were greater in PDs versus controls during SFT (P < 0.05). Also, AFT, but not SFT, detected a difference between the MA and LA hands of PDs (P < 0.01).

**Conclusions:**

Unimanual, repetitive alternating finger tapping brings out more arrhythmokinesis compared to bimanual, single finger tapping in PDs but not in controls. Arrhythmokinesis during unimanual, alternating finger tapping captured a significant difference between both the MA and LA hands of PD subjects and controls, whereas that during a bimanual, single finger tapping task only distinguished between the MA hand and controls. Arrhythmokinesis underlies freezing of gait and may also underlie the freezing behavior documented in fine motor control if studied using a unimanual alternating finger tapping task.

## Background

Wertham used the term “arrhythmokinesis” to describe the variability in repetitive movements in cerebellar diseases [1]. Arrhythmokinesis has subsequently been revealed in many studies of repetitive movement in Parkinson’s disease (PD) and has been shown to be a useful marker of overall disease severity and of the effectiveness of medication and deep brain stimulation [2-10].

Arrhythmokinesis of stride duration during walking and stepping in place tasks has been shown to correlate with self-reported freezing of gait (FOG) severity, and has been suggested to be a useful marker of FOG [11-13]. Freezing behavior has also been demonstrated in repetitive finger and upper extremity movements [9,14-17]. In studies using single finger flexion-extension movements, alternating between hands, the number of freezing episodes correlated with self-reported (FOG) severity but arrhythmokinesis was not observed during the task [14-17]. The authors concluded that arrhythmokinesis of upper extremity movements may not be a useful marker of freezing behavior as had been reported for FOG. However, other reports have suggested that more complex upper limb tasks may be required to detect arrhythmokinesis in upper limb movements [17]. Using a unimanual alternating finger tapping task we have reported both freezing episodes and arrhythmokinesis [9,10]. This inspired us to investigate whether a more complex task was necessary to reveal arrhythmokinesis in upper extremity movements. In this study, we measure arrhythmokinesis in both amplitude and frequency in a repetitive unimanual alternating finger tapping (AFT) task and compare that to the same measures in a repetitive bimanual single finger tapping, alternating between hands (SFT) task in PD subjects, off medication, and in an age-matched group of healthy subjects.

## Methods

### Subjects and clinical evaluation

Sixteen subjects (twelve men) with idiopathic Parkinson’s disease (PD) were recruited from the Stanford Movement Disorders Clinic and consented to participate in the study, which was approved by the Stanford Institutional Review Board. A fellowship-trained Movement Disorders specialist confirmed the diagnosis in each subject. The age at evaluation was 68.9 ± 9.02 years, and the disease duration from diagnosis was 8.2 ± 5.64 years. Patients reported the body side that was more affected by PD, which was confirmed by the UPDRS III.

Twenty-four age-matched control subjects (57 ± 7.98 years, six men) consented and participated in the study. Control subjects were screened for any coexisting neurological or medical disorder using a comprehensive questionnaire. There were more men in the PD group than in the control group, which would tend to bias the results towards more rhythmic tapping in PD subjects, as men may tap with greater regularity than women [18].

The PD subjects were tested in the off therapy state. Thirteen subjects had not undergone deep brain stimulation (DBS) surgery and three subjects had the DBS system implanted but were tested prior to initial activation of the DBS system. All subjects withheld long- and short-acting dopaminergic medication for >24 and >12 hours respectively prior to testing [10].

### Quantitative DigitoGraphy (QDG)

**Figure 1:**
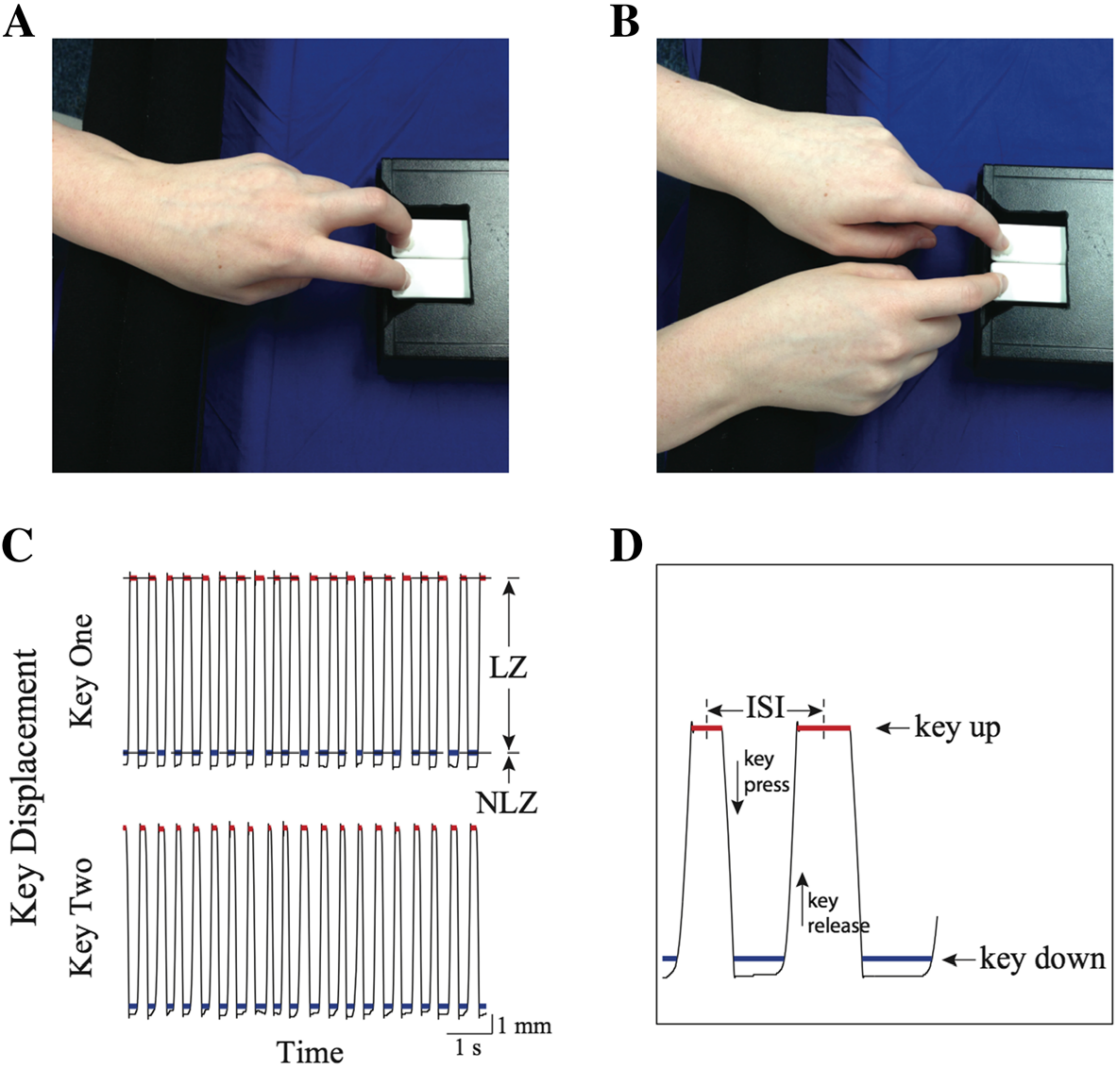
**Data acquisition on customized engineered keyboard.** Index and middle finger placement on the engineered keyboard for **A**: unimanual, repetitive alternating finger tapping (AFT) and **B**: repetitive single finger, alternating hand tapping (SFT). **C**: schematic diagram of key displacement zones. LZ = linear zone, NLZ = Non-linear zone, **D**: higher magnification diagram of the key displacement epochs, ISI = inter-strike interval.

Subjects sat on an armless chair with the elbow flexed at approximately 90 degrees and the wrist resting on a pad at the same level as the keys of a customized engineered, MRI compatible keyboard, which allows for precise measurement of amplitude and timing of finger tapping, Figure 1. We have called this technology Quantitative DigitoGraphy (QDG) [9,10].

Subjects performed the AFT task, Figure 1A and the SFT task, Figure 1B, in a predetermined randomized order. In the AFT task, subjects placed the index and middle finger on each key with the other hand resting on the lap. In the SFT task, subjects placed the right and left index finger on each key. The other fingers did not touch the surrounding keyboard or tabletop area. In both tasks, subjects were instructed to tap each key in an alternating pattern as fast and as regularly as possible for 30 seconds, starting and stopping only when they heard an auditory cue, while maintaining the alternating sequence and keeping the wrist stationary. Participants were instructed to attempt to press and release the keys completely. The same instructions were given to each subject.

No external pacing or cueing was provided. Subjects performed the tasks without visual (eyes closed) or auditory feedback. The keys do not produce audible notes and subjects wore headphones through which static “white noise” was transmitted to mask the sound of the finger striking the key. Each subject had a short period of practice before the test began, and all trials were videotaped; the video of the fingers was time-stamped and synchronized with the data.

### Data acquisition and analysis: customized engineered keyboard and kinematic algorithm

An engineered keyboard was used to acquire the finger tapping movement, Figure 1. The keyboard was designed to produce an analog output voltage signal proportional to the displacement of the key. The key displacement was linearly related to the output voltage signal with a resolution of 62.5 μm per 40 mV. For key displacements less than 9 mm, the keyboard operated in a linear zone (LZ), Figure 1C. Systematic analysis of the keyboard mechanics revealed that near the base of the key displacement, the key reached a compliant mechanical stop. When the finger pressed down further, the extra displacements were non-linearly related to the output voltage signal. We defined this range of the key displacement as the “nonlinear zone” (NLZ), Figure 1C.

A customized detection algorithm, written in MATLAB (version 8.2, The Mathworks, Inc., Natick, MA, USA), was used to determine specific states in the cycle of finger tapping movement. Each cycle of finger tapping was divided into four epochs, Figure 1D. The “key up” epoch was defined as the period during which the finger changed direction from an upward movement to a downward movement. The “key down” epoch was the time when the finger changed direction from a downward movement to an upward one. The “key press” epoch was the period during which the finger was moving downwards. Finally, the “key release” epoch was the period during which the finger traveled upwards.

The inter-strike interval (ISI), the time to complete one cycle of finger tapping, was calculated from the midpoint of one key up epoch to the midpoint of the following key up epoch, Figure 1D. The variability of key tapping frequency was measured using the coefficient of variation (the standard deviation (SD) divided by the mean) of the inter-strike interval (CV_ISI_) and was reported as a percentage.

The key strike amplitude was calculated for key displacements in the linear zone of the keyboard. The amplitude of a key press or release was defined as the distance (mm) the key travelled during key press or release epochs respectively. The maximum amplitude for a complete key press or release in the linear zone of the keyboard was 9 mm. For each subject, the average of all the key press and key release epochs was used as the amplitude outcome metric. The variability of amplitude was calculated using the coefficient of variation (CV_AMP_).

### Statistics

The statistical software R (R Core Team, 2013) and lme4 (Bates, Maechler & Bolker, 2012) were used to perform a linear mixed effects analysis of the relationship between group type and test type. Group type (PD-most affected hand, PD- less affected hand, Control- dominant hand, and Control- non-dominant hand) and test type (AFT, SFT) with all interaction terms were fixed effects, and subject number was a random effect. The model included an intercept term. Visual inspection of residual plots did not reveal any obvious deviations from assumptions of homoscedasticity or normality. P values were obtained using the multcomp package in R (Bretz, Hothom, Westfall, 2010) with a Tukey correction for multiple comparisons.

## Results

**Figure 2:**
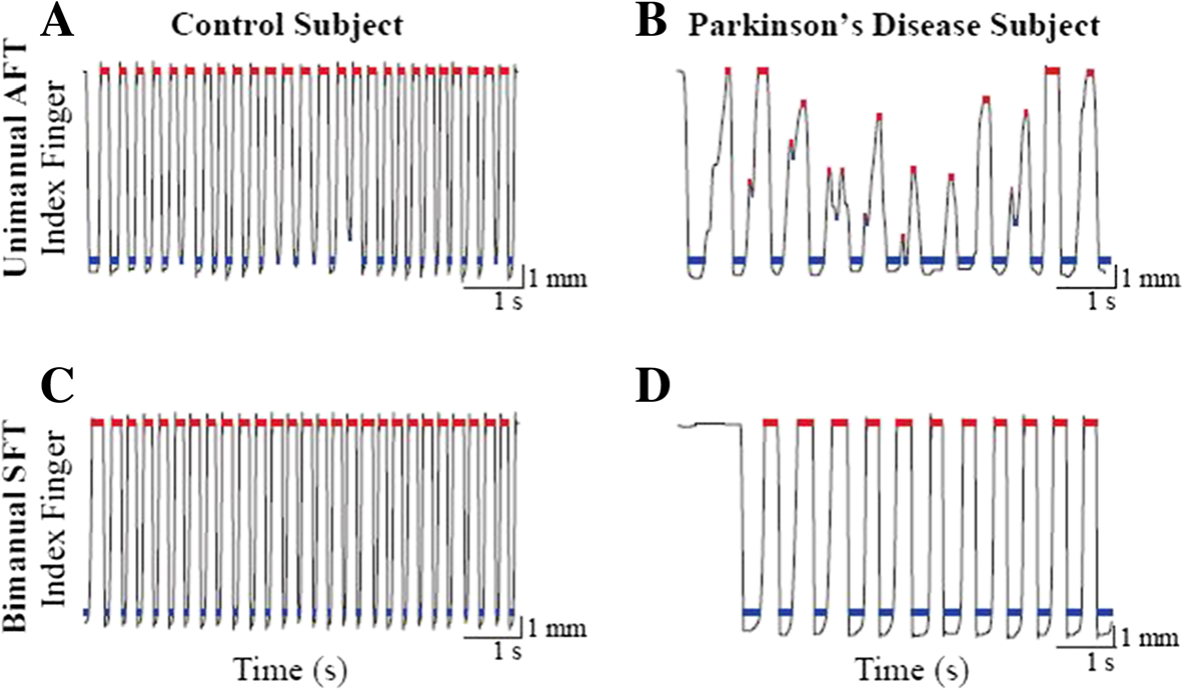
**Representative finger tapping data during AFT and SFT tasks.** QDG data from seven seconds of index finger tapping in the AFT and SFT tasks for a control subject (NDH, **A** and **C**) and a representative PD subject (MA side, **B** and **D**).

The group’s off medication UPDRS III (motor) score ± SD was 35.5 ± 11.34, and the lateralized upper extremity scores (rest tremor, postural tremor, rigidity, finger tapping, hand movements, pronation-supination of the hands) were 10.19 ± 4.74 and 6.2 ± 3.16 in the more affected (MA) and less affected (LA) sides, respectively. Figure 2 demonstrates the QDG data from seven seconds of index finger tapping in the unimanual AFT and bimanual SFT, alternating between hands, tasks from a control subject’s non-dominant hand (NDH), Figure 2A and C respectively, and from the MA hand of a PD subject off medication, Figure 2B and D respectively.

The control subject performed the AFT task with fast, regular, and mostly full amplitude key presses, while the PD subject performed AFT with arrhythmic partial key presses, Figure 2A and B, respectively. In contrast, both subjects performed SFT with a regular rhythm and amplitude, although the PD subject tapped more slowly than the control, Figure 2C and D, respectively.

**Figure 3:**
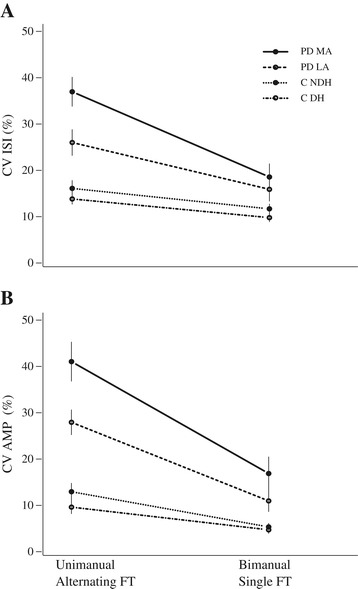
**AFT captures greater arrhythmokinesis than SFT in PD subjects but not controls.** Coefficient of variation (CV) of ISI **(A)** and amplitude **(B)** for the control subjects’ DH and NDH and PD subjects’ MA and LA sides during AFT and SFT.

PD subjects, but not controls, exhibited more irregular tapping during the AFT task compared to the SFT task, Table 1 and Figure 3.

**Table 1:** **AFT captures greater arrhythmokinesis than SFT in PDs but not controls**

	**Unimanual AFT**	**Bimanual SFT**	**P Value**
	**CV**_**Amp**_ **(25-75% range) or (SD)**	**CV**_**Amp**_ **(25-75% range) or (SD)**	
PD MA	38.6 (16.9)	14.5 (14.3)	P < 0.001
PD LA	25.5 (10.7)	8.55 (9.20)	P < 0.001
Control DH	7.21 (6.76)	2.31 (3.75)	NS
Control NDH	10.5 (8.94)	2.94 (3.82)	NS
	**CV**_**ISI**_ **(25-75% range) or (SD)**	**CV**_**ISI**_ **(25-75% range) or (SD)**	
PD MA	37.0 (12.5)	18.6 (11.4)	P < 0.001
PD LA	26.1 (15.9-30.3)	12.6 (9.15-18.5)	P < 0.05
Control DH	13.8 (5.4)	9.79 (4.21)	NS
Control NDH	14.28 (10.04-17.98)	10.45 (7.61-16.21)	NS

Values are reported as percentages.

This was evident for both the amplitude and timing (frequency) of tapping among PD subjects in the MA and LA hands, Table 1. There was no difference in variability of amplitude or timing during AFT versus SFT in the DH and NDH of controls. Tapping of the MA hand was more irregular than that of the LA hand in PDs during AFT (P < 0.01 for CV_ISI,_ P < 0.001 for CV_AMP_) but not SFT. There was no difference in variability of amplitude or timing between the DH and NDH of controls in either task.

The performance of unimanual AFT was more irregular in both the PD MA and LA hands compared to control DH and NDH for both amplitude (CV_AMP_, P < 0.001, for all) and frequency (CV_ISI_, P = 0.01 for LA vs. NDH, P < 0.001 for all others, Figure 3). Comparing the performance of bimanual SFT between PD subjects and controls: CV_AMP_ was greater in the PD MA hand compared to both control DH and NDH (P < 0.01 for both) and CV_ISI_ was greater in the PD MA hand compared to the control DH (P < 0.05). The CV_AMP_ and CV_ISI_ of bimanual SFT were not different between the PD LA hand and control DH or NDH.

## Discussion

This study has shown that arrhythmokinesis was worse during a repetitive unimanual alternating finger tapping (AFT) task than during a repetitive bimanual single finger tapping, alternating hand (SFT) task in both the more affected and less affected hands in PD subjects, off medication. There was no difference in the regularity of the amplitude or the frequency of tapping between tasks in control subjects. Arrhythmokinesis in PD MA and LA hands was worse in the unimanual AFT compared to the performance of controls DH and NDH, whereas only the PD MA hand was more irregular than the control NDH (amplitude) and DH (amplitude and frequency) in the bimanual SFT task.

Arrhythmokinesis in upper extremity movements in Parkinson’s disease has been shown to be a useful marker of overall disease severity and of the effectiveness of medication and deep brain stimulation [2,7-9]. Arrhythmokinesis of stride duration and amplitude is a reliable marker of freezing of gait (FOG) and is related to cognitive deficits, which may also contribute to FOG [11,12,14,19-23]. However, a study comparing freezing behavior in repetitive upper extremity movements to FOG failed to reveal evidence of arrhythmokinesis even though they did demonstrate freezing episodes that correlated with FOG [14,17,24]. The findings of the current study support the hypothesis that a more complex task than single finger tapping, alternating between hands may be necessary to demonstrate arrhythmokinesis in upper extremity and specifically finger movements.

Coordination of alternating movements of adjacent digits on one hand appears to be more challenging to the brain than the coordination of a simple movement of one digit per hand, alternating between hands. Functional imaging (fMRI) has demonstrated that a similar bihemispheric motor network was activated during bimanual and unimanual finger movements in normal subjects [25]. However, there was increased activation in several regions during the unimanual alternating finger movement compared to the bimanual single finger movement. In a study comparing unimanual single and alternating finger tapping in PD, only alternating finger tapping with activation of the contralateral limb correlated with the striatal 6-Fluoro-L-dopa PET uptake [26]. Thus, it may be more challenging for the impaired sensorimotor network in PD to maintain the regularity of an alternating, adjacent finger tapping task of one hand than to maintain regularity in an alternating hand movement with single finger tapping.

Previous studies have shown that the basal ganglia is involved in motor and perceptual timing, and that PD subjects are impaired in repetitive motor timing [27]. In addition, tasks with increased cognitive demands show greater impairment in PD subjects [28]. Therefore, it is possible that the unimanual AFT task captured greater arrhythmokinesis as it involves increased cognitive load compared to single finger tapping. It has also been proposed that the bihemispheric activation of motor circuits during bimanual tasks may facilitate movement of the more affected side [29]. Thus during bimanual SFT, it is possible that the less affected limb is able to maintain a more normal rhythm and “drive” the more affected limb to keep time.

The actual value of the CV that is used to differentiate regular from irregular behavior differs among motor tasks in PD and control subjects, and this may have biological significance. In several studies of FOG, the CVs reported for stride length or duration during walking and stepping tasks were consistently around 3% for non-freezers and controls and 6% for freezers [11-13,21,30]. In this study of unimanual AFT the CVs were greater than 7% for control subjects and greater than 25% for PD subjects and are consistent with our previous AFT studies using a MIDI-interfaced keyboard [10].

The explanation for the difference in CVs of unimanual alternating finger tapping versus those of stride duration may be similar to the explanation for the difference in variability between stride length and duration (2-5%) versus stride width and double support time in healthy subjects (17-27%) [31]. It was postulated that stride length and duration reflect gait patterning, which requires a high degree of consistency, while stride width and double support time are balance control mechanisms well-suite for fine control with a larger degree of variability. Thus higher values of the CVs in unimanual AFT in both control and PD subjects support a motor control mechanism more suited for variability and fine-tuning than for consistency.

## Conclusions

Arrhythmokinesis, the variability in repetitive movements, may be regarded as a cardinal motor sign in Parkinson’s disease. In this study, unimanual AFT captured greater arrhythmokinesis than bimanual SFT, in PD subjects. This difference was not seen in control subjects. Unimanual AFT also captured a significant difference in arrhythmokinesis between the PD MA and LA hands and controls’ DH and NDH whereas bimanual SFT only distinguished between PD MA hand and controls’ NDH (amplitude) and DH (amplitude and frequency). Arrhythmokinesis of stride duration is widely used as a marker of FOG. A study of bimanual single finger movements alternating between hands demonstrated freezing episodes, but not arrhythmokinesis, leading to the conclusion that arrhythmokinesis is not a marker of freezing behavior in upper extremity movements. The results of this study demonstrate that arrhythmokinesis in fine motor control may be evident if investigated during more complex finger movement tasks, such as a unimanual alternating adjacent finger tapping task. It remains to be seen if this correlates with FOG metrics.

## Abbreviations

PD: Parkinson’s disease
AFT: Alternating finger tapping
SFT: Single finger tapping
MA: More affected side
LA: Less affected side
UPDRS: Unified Parkinson’s Disease Rating Scale
FOG: Freezing of gait
QDG: Quantitative digitography
LZ: Linear zone
NLZ: Non-linear zone
ISI: Inter-strike interval
NDH: Non-dominant hand
DH: Dominant hand
